# First-year medical students’ perceptions of a self-regulated learning-informed intervention: an exploratory study

**DOI:** 10.1186/s12909-022-03908-4

**Published:** 2022-11-29

**Authors:** Taylor Boyd, Henrike Besche, Richard Goldhammer, Afaf Alblooshi, Bradley I. Coleman

**Affiliations:** Harvard Medical School, 25 Shattuck St, Boston, MA 02115 USA

**Keywords:** Self-regulated learning, Reflection, Goal setting

## Abstract

**Background:**

Students with developed self-regulated learning (SRL) skills demonstrate an ability to set individualized educational goals, select optimal learning strategies for reaching these goals, and reflect on overall progress. The primary aims of this study were to investigate first-year medical students’ perceived utility of a self-regulated learning-informed intervention and to assess the impact of its implementation on students’ intended use of SRL throughout medical school.

**Methods:**

A two-part educational intervention focused on SRL skill development was carried out at Harvard Medical School during the start of the 2021 academic year. For the first component of the intervention, 169 first-year medical students engaged in an interactive class session structured around SRL concept videos, a brief lecture, small group discussions and individual reflection. Students completed pre- and post-intervention surveys which inquired about learners’ current and anticipated application of SRL skills. During the second component of the intervention, 15 first-year medical students participated in a set of one-on-one academic SRL coaching sessions. All coaching participants completed follow-up semi-structured interviews.

**Results:**

A statistically significant increase was observed between students’ use of skills in all domains of self-regulated learning prior to the intervention and their anticipated use of these skills following the intervention. Prior to the intervention, 60.1% (*n* = 92) of students reported utilizing evidence-based learning strategies, compared to 92.8% (*n* = 142) of students (*p* < 0.001) who anticipated applying this SRL skills at the completion of the classroom session. Six core themes emerged from qualitative analysis of the post-intervention survey including learning plan development, accountability and progress tracking, goals for growth, engagement through active learning, routine reflection, and adapting to the curriculum.

**Conclusions:**

Both classroom-based learning sessions and one-on-one academic coaching programs are feasible approaches for encouraging the use of self-regulated learning techniques in the preclinical setting.

**Supplementary Information:**

The online version contains supplementary material available at 10.1186/s12909-022-03908-4.

## Background

First-year students may enter medical school with significant variation in academic strengths and areas for growth [1]. Students’ success in the preclinical setting often requires acquisition and application of a considerable amount of complex clinical knowledge and even learners well-accustomed to meeting the demands of rigorous curricular standards may face new educational challenges [2]. In settings where innovative formats of learning, such as case-based learning (CBL), problem-based learning (PBL), and other formats of collaborative education are implemented, potential learning challenges may be amplified during the early stages of medical school [3]. In collaborative learning, students prepare independently prior to class and utilize time with peers to solve clinical problems. Throughout this active format for small group learning, students receive immediate feedback on their understanding of a topic based on the accuracy of their responses and problem-solving abilities. The educational benefit resulting from participation in active, team-based classroom learning is often influenced by students’ ability to continuously reassess understanding and adapt learning processes based on external feedback [4–8].

In self-regulated learning, students engage in a cycle of setting specific goals, considering and personalizing their learning strategies, and finally reflecting on and adapting behaviors to optimize educational achievement [9, 10]. The application of self-regulated learning strategies among medical students may be associated with academic achievement in the preclinical setting [11].

While there is increasing evidence of the benefit of self-regulated learning throughout preclinical training, incorporating protected time for students to fully participate in reflection and to assess new approaches to learning may be a challenge [12, 13]. Prior studies among first and second-year medical students have investigated the implementation of interventions such as learning dashboards, planning, coaching, and reflection activities on self-regulated learning in the preclinical setting with variable results [12, 14, 15]. However there has been little research specifically in the context of CBL and PBL, designed to enhance the development of SRL skills or to motivate the application of these strategies among medical students using coaching and classroom-based interventions [12, 14, 15]. In this study, an SRL intervention consisting of both a classroom session and a one-on-one coaching program was implemented early in the first semester of students’ first year of medical school. The primary aims of this study were to 1) explore learners’ perceptions about the utility of a self-regulated learning-inspired intervention and 2) assess the impact of an SRL intervention on medical students’ expected use of goal setting, evidence-based learning strategies, and reflection early in medical school.

## Methods

This project was reviewed and approved by the Harvard Medical School Program in Medical Education Scholarship Review Committee and was exempt from formal IRB review. The study population included 169 students enrolled in their first year of preclinical training at Harvard Medical School. Among first-year medical students who participated in the study there were differences in educational backgrounds. Some students had science-based undergraduate majors such as biology or chemistry, while other held degrees in humanities, language, math, art, or other areas of study.

The intervention was piloted in the setting of a flipped classroom curriculum which necessitates student engagement in several hours of dedicated preparatory work prior to class sessions. The preclinical curriculum in this format also focuses heavily on case-based collaborative learning, in which a problem or scenario is used to stimulate the acquisition of knowledge amongst learners within a small group. For many students, case-cased learning is a new format of education. More traditional, lecture-based learning may place greater emphasis on memorization and routine performance on exams. This intervention was designed to aid students in the application of self-regulated learning in the context of case-based learning, a format of education which requires perspective and reflection to facilitate growth and improvement. The intervention design was based on the Social-Cognitive Theory as proposed by Zimmerman et al. [16]. This model incorporates three stages of self-regulated learning, including 1) the planning and forethought phase which involves setting goals, 2) the learning and performance phase which encompasses the implementation and experimentation of learning strategies and 3) the evaluation and self-regulation phase, consisting of reflection on performance [16]. For the purposes of the intervention, and to make the steps of self-regulated learning more relatable and actionable for medical students, the three phases were simplified to represent the core principles of self-regulated learning including 1) goal setting, 2) applying evidence-based learning strategies, and 3) reflection. This simplified model has also been used in prior studies involving self-regulated learning among medical students [17]. In this study, evidence-based learning strategies refers to methods for learning which have been shown to improve long-term retention of learned material including self-quizzing, consolidation, and interleaving [18].

Research has suggested beneficial effects of student engagement in the cyclical process of self-regulated learning delivered through a diversity of formats. The majority of prior studies have involved the implementation of coaching and instructional small or large group guidance. There have been notable differences in measured outcomes depending on SRL intervention design [12, 15, 19]. Incorporating the intervention utilizing both the large group format as well as in an individualized coaching setting, allowed for the assessment and comparison of students’ experiences in both settings and formats.

### Intervention 1: classroom session methods

The classroom session was carried out as a quality improvement project. Anecdotal evidence from students suggested a need for enhanced education regarding self-regulated learning. Professional development content centered around self-regulated learning was developed, and all 169 first-year students were invited to participate in the session regardless of prior performance and experience with self-regulated learning. The initial component of the intervention took place during medical students’ first semester of the preclinical curriculum. Prior to attending the in-class session, students watched a series of four brief videos designed to introduce the concept of SRL, as well as the different SRL domains including goal setting, evidence-based learning strategies, and reflection.

Students attended a 90-minute, faculty-led class session on SRL skill development and application. The session consisted of a brief lecture and small group discussions based on the SRL concepts introduced in the videos. The class also included time for individual reflection and goal setting. All students were provided with a personal journal in which to address prompts for reflection. As a part of the classroom intervention, students filled out two questionnaires (see Additional file 1). The first questionnaire was completed after watching the videos, prior to the classroom session. This pre-class survey included questions regarding students’ current use of SRL skills and inquired about how one might advise a fellow medical student to apply self-regulated learning and potential barriers they anticipate their colleague may encounter. Following the class session, students completed a second survey and responded to questions regarding anticipated use of skills in SRL, potential barriers to future application of skills and specific examples of how they may incorporate the use of these strategies into their own routine.

### Intervention 2: academic coaching sessions methods

The academic coaching intervention was carried out as a pilot study and session content was delivered during the sessions for a total of 15 first-year medical students. The purpose of the pilot was to determine feasibility, required material, and resources necessary for a potential larger scale intervention. All first-year medical students were invited via email to participate in a series of two individualized academic coaching sessions led by one of the medical school’s learning specialists (RG), and 15 students were randomly selected to participate. Each meeting with the academic coach was 40 minutes, with sessions spaced approximately 2 weeks apart.

The initial coaching session (see Additional file 1 for coaching guide) consisted of four primary objectives including 1) establishment of a relationship between the coach and coachee, 2) development of an individualized learning plan and/or goals based on student-reported strengths and areas for growth 3) discussion of evidence-based learning strategies and key psychological principles in learning (i.e. growth mindset) and finally 4) real-time practice using new learning strategies based on content and examples extracted from the first-year basic science curriculum. The second coaching session included 1) a discussion of progress and/or challenges in implementation of the learning plan, 2) provision of objective feedback and suggested solutions to learning challenges from the learning coach, and 3) revision and adjustment of learning goals for the remainder of the semester. Following the second coaching session, each student took part in a virtual interview. A broad, open-ended semi-structured interview protocol was used to guide the discussion (see Additional file 1). One test interview was conducted.

### Data analysis

Quantitative pre and post survey data from the classroom intervention was analyzed using the statistical software STATA Version 16 (Stata Corp, College Station, Texas. StataCorp. 2019). Chi-square tests were used to determine statistical significance of differences between students’ pre and post survey responses.

Interviews from the academic coaching session were video recorded, transcribed verbatim, and de-identified. Both data from the transcribed interview responses and qualitative short answer survey responses from the classroom session were stored and organized using Dedoose Version 9.0.46 (Sociocultural Research Consultants, Los Angeles, California). Two authors (TB and AA) who did not engage with students in the classroom or coaching sessions carried out the qualitative analysis. Students’ short responses from the surveys and transcribed interviews were read through in entirety and an inductive approach was employed using the Framework Method for qualitative analysis [20]. Memoing was completed, followed by open coding and codebook development. Descriptions and illustrative quotes for every code were discussed and discrepancies resolved prior to application of codes to the entire data set. Intercoder reliability was established through coding identical texts and resolving differences of opinion through discussion. Final themes were determined through an iterative process of constructing level 1 and level 2 categories from coded excerpts. Coders met throughout the coding process to ensure full agreement and discussion of uncertainties in coding. Emerging themes were discussed with the entire research team twice during committee meetings.

## Results

### Part 1: classroom intervention survey results

**Fig. 1:**
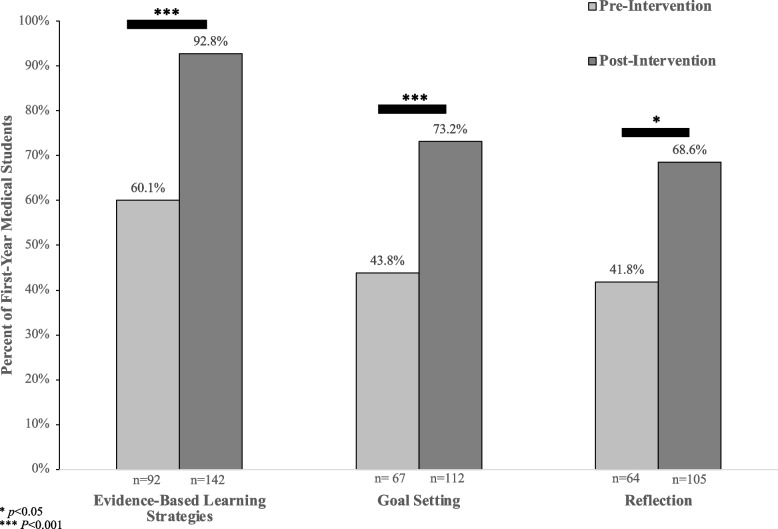
First-Year Medical Students’ Pre-Intervention Use and Post-Intervention Anticipated Use of Self-Regulated Learning Skills on a Weekly Basis. Bar graph showing the percent of the total number of participating students (*n* = 153), who reported using each of the self-regulated learning (SRL) skills at least once a week, prior to the classroom intervention (light gray bar) compared to the number of students who anticipated using each of the SRL skills once a week at the conclusion of the in-class intervention (dark gray bar). There was a statistically significant difference in pre and post survey responses for all domains of self-regulated learning

**Fig. 2:**
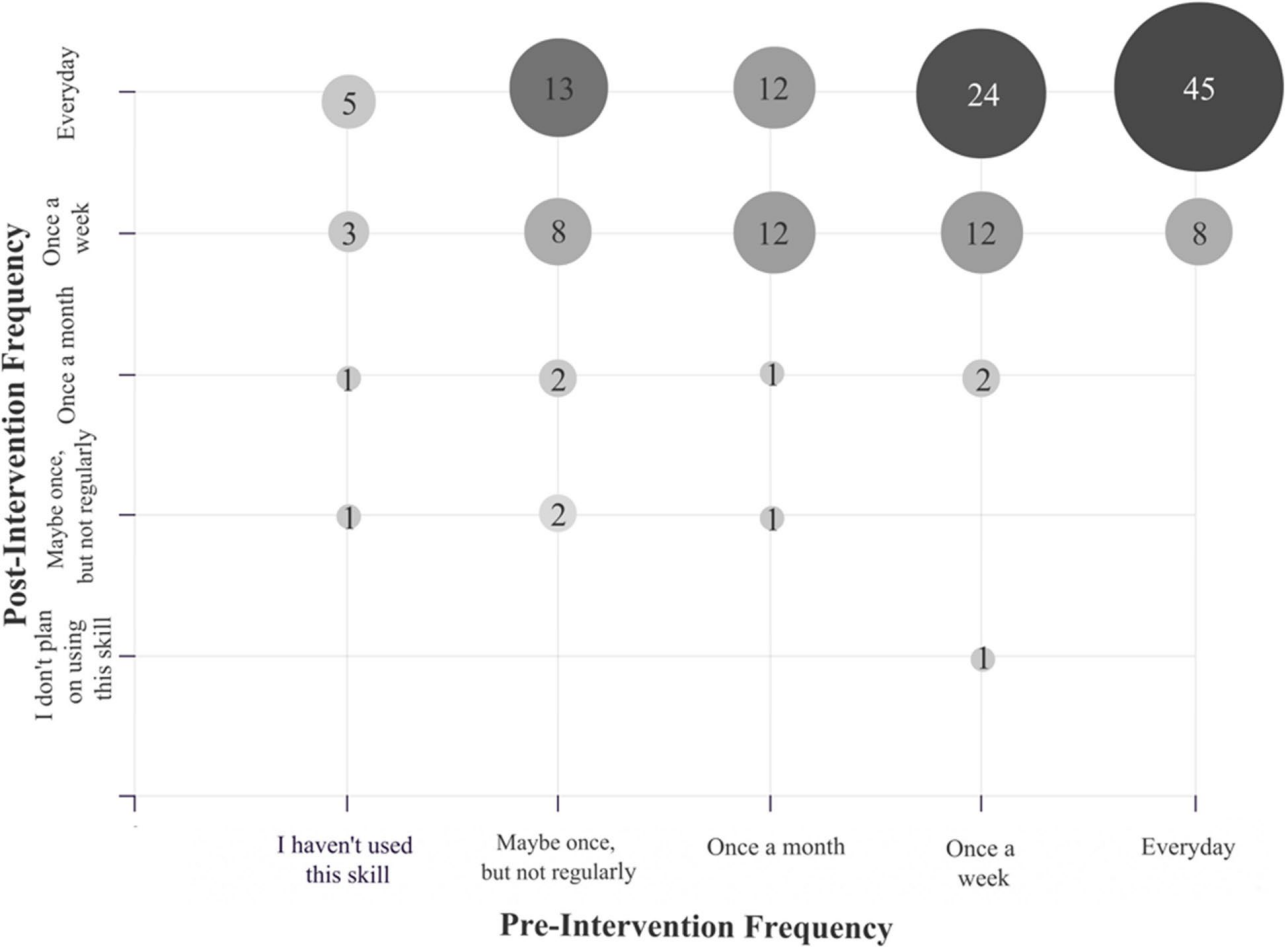
First-Year Medical Students’ Pre-Intervention Use and Post-Intervention Anticipated Use of Evidence-Based Learning Strategies. Bubble plot depicting the frequency of first-year medical students’ reported pre-session use of evidence-based learning strategies and post-session anticipated use of evidence-based learning strategies (*n* = 153). The number at the center of each bubble represents how many students indicated using evidence-based learning strategies at each frequency before the intervention, and their anticipated frequency of use of evidence-based learning strategies after the intervention. Bubble shade and size correlate with the number of students in each group, with the larger and darker shades representing a greater the number of students

In total, 153 out of 169 students completed both the pre and post surveys, with a response rate of 90.5%. A statistically significant increase was observed between students’ use of skills in all domains of self-regulated learning prior to the intervention and their anticipated use of these skills after the intervention (Fig. 1). Students who expected to use evidence-based learning skills every day, once a week or once a month after the intervention varied in their initial use of this SRL domain, ranging from never having previously utilized evidence-based learning strategies, to already applying these skills daily (Fig. 2).

In total 1224 independent qualitative student responses from the classroom intervention pre- and post-survey were coded and analyzed (survey prompts located in Additional file 1). Based on the post-intervention questionnaires, first-year medical students anticipated using a wide variety of SRL skills during their first semester of medical school, and six themes emerged from qualitative analysis. The primary themes included learning plan development, accountability and progress tracking, goals for growth, engagement through active learning, routine reflection, and adapting to the curriculum (Table 1).

**Table 1:** Themes in First-Year Medical Students’ Planned Application of Self-Regulated Learning Skills

Theme:	Description:	Illustrative Quote:
**Accountability and Progress Tracking**	Milestones such as mid or end-of-course assessments may be used to check in on progress, build on previous goals and target areas of weakness in knowledge and learning process. Students strive to obtain and apply constructive, objective external feedback. Learners look to peers, mentors, and professors as a source of accountability.	“I could develop and check-in with my goals in conjunction with the periodic meetings I will have with my society advisor. These meetings could serve as useful benchmarks for progress towards my goals and could help me bring forward questions I may have or resources I need to achieve my goals.”
**Goals for Growth**	Students hope to set and achieve professional, academic, and personal goals, with the most important components of the goal achievement process including: (1) Breaking down larger goals into smaller goals (2) Celebrating progress and achievements (3) Maintaining a growth mindset	“I plan to set smaller goals for each week in terms of the classroom material I need to do and to achieve the larger goals surrounding research and my medical career. Then once a month approximately, I would like to adjust the larger goals and ensure I am on the path I would like to be.” “I always reflect on how I did a particular learning exercise and how I can improve in the future. I try to have a growth mindset throughout my learning process.”
**Engagement Through Active Learning**	Students anticipate using a variety of learning skills, tools, and resources throughout the semester specifically citing: (1) Active recall (2) Self-quizzing (3) Spacing (4) Consolidation (5) Diagram and table creation (6) Paraphrasing (7) Interleaving (8) Summarization (9) Elaboration (10) Talking through processes aloud (11) Developing questions (12) Using flash cards (13) Engaging with efficient study groups Students are mindful of resorting to less efficient, but potentially more comfortable learning habits such as re-reading, re-watching videos, and passively taking notes.	“I have already begun to change the way I approach prep as a result of the session. I am trying to really push myself in efficiency and challenge how I am absorbing the material. I realize a lot of what I was doing before felt secure and productive, as I would write down what the videos were saying but in terms of actual recall and encoding, the information was not as effective.” “I love to turn notes into pictures and consolidated tables. I can attempt to convert my lengthier notes into a series of graphics that organize the concepts. It will probably take extra time investment up front, but I suspect it will pay off on the long-term.”
**Routine Reflection**	Students recognize a benefit in making reflection and goal setting a part of a regular learning routine and plan to commit to daily, weekly or monthly reflection. Learners see value in physically writing out reflections in a journal or portfolio as opposed to solely reflecting with thoughts. Many students plan to use the journal provided to them in class for reflection.	“Ongoing reflection will ensure my personal and professional growth. I am planning to utilize the notebook that we all received during last week’s session to jot down the most important moments of each month.” “At the end of the week, I will take 15 minutes to think back to different parts of my learning routine - from efficiency, to retention, to my ability to see the bigger picture. This helps me consolidate my learning path, while also pushing myself to set goals for future steps I could take to enhance my learning.”
**Adapting to the Curriculum**	Students aim to use strategies already incorporated into the curriculum to their advantage (e.g., interleaving, self-quizzing).	“Evidence-based learning is something that we will need to continue throughout our profession so it’s important to start to learn now. Interleaving, retrieval practice and elaboration are all important learning strategies. These are all nicely incorporated into the way [basic science course] is set up. So as long as we follow the design, we should naturally use all of these strategies.”
**Learning Plan Development**	To achieve learning goals, students anticipate creating plans for learning. Plans may outline specific learning skills which will be employed, content which will be covered, estimated amount of time to be spent and/or the environment where learning and studying will take place. Students use reflection and self-questioning to identify areas for growth which serve as the focus of learning plans.	“I want to establish a solid routine of preparatory work, going to class, consolidation, and review. In each of these, I will actively engage in the material and work on recall instead of passively interacting with the material and/or being prompted to remember.”

The first column of Table 1 shows six themes derived from the qualitative analysis of 612 free text responses from first-year medical students (*n* = 153) who completed the post-class survey. The second column includes a description of each theme, and the third column reports a representative student quote to further illustrate the theme and description. Students responded to questions inquiring about if and how they plan to incorporate evidence-based learning, goal setting, and reflection into their learning routine (see Additional file 1 for post-survey questions)

### Part 2: coaching intervention results

Approximately 20% (*n* = 33) of students who participated in the classroom session responded to an email invitation indicating an interest to continue engaging in SRL skill development through participation in academic coaching. A cohort of fifteen learners were selected via random sample from this group. Analysis of post-coaching interviews revealed a total of 8 themes. Two themes related to students’ motivation for participating in the coaching program, three related to the overall coaching experience, and the final three were associated with the benefits of working with an academic coach.

### Motivation for participation in coaching: themes 1 & 2

#### Theme #1: adapting to learning in medical school

The transition to learning in medical school is a period of academic adjustment, often associated with uncertainty as a result the introduction of new methods for active, team-based classroom learning in addition to the large volume of information presented. Students are motivated to identify learning support, resources and strategies which help improve long-term retention and application of knowledge.

• “I’ve been a student for many years now, I feel I know how to study but this is a little bit different in medical school. Here, it is a lot of volume that is very diverse and my methods were not necessarily as effective. I care a little bit more about long-term retention and feel like a lot of the stuff I learn here will be important later on, so I want to learn in a better way. That's why I signed up, I thought it would be helpful to just go through this with someone.”

• “Coming to med school, the way classes are setup is very different than undergrad … I’m used to cramming a lot. I wanted to like have someone talk me through that and it ended up being really helpful I think. Sometimes you just need a little push, well I needed a push to be more efficient.”

#### Theme #2: evolving as a strategic learner

Students acknowledge that a career in medicine requires continuous improvement in learning skills and that small changes to improve learning efficacy can make a difference in the long-term.

• “I feel like there's always room for improvement. Even though I was happy with the way that I was studying, if I can be more efficient, then why not? Things are going to definitely get more difficult or challenging so this is the best time for me to learn how to study more efficiently.”

• “My initial motivation was largely driven because I knew that the way I was studying was not sustainable and having a career as a physician is a lifelong journey of learning. If I feel burned out from the beginning, I know that I need to change something.”

### Overall coaching experience: themes 3-5

#### Theme #3: learner led reflection

Students appreciated aspects of the coaching experience which were guided primarily by their own reflection. Students viewed both the individualization of each session and the coach’s role in supporting and respecting their learning decisions, while providing specific feedback favorably,

• “I think what's really important and what was evident in these sessions was that [academic coach] really asked me to first share about what I’ve been doing and how I think it's going, and whether it's working or not. And I think that reflection is important, because I can do that by myself but it's very powerful when you have someone else on the other side.”

#### Theme #4: reassurance and validation from the coaching process

Students felt validation from discussing and normalizing feelings of doubt and discomfort associated with adapting to learning and studying in medical school.

• “…It’s a new way to learn, not like going to lecture and then like cramming before the exam. It’s like a real continuous learning process that I think it does take some adaptation to get accustomed. These coaching sessions are just like a time to chat about that, until my worries are out and it’s sort of like here is what a professional would have to say about it.”

#### Theme #5: time and space for reflection

Students recognize the importance of reflection but find it challenging to identify the time to incorporate this SRL practice. Coaching sessions provided space, structure, and support to reflect on areas for improvement.

• “It is easy to subconsciously reflect on things, it's harder in the pace in med school to sit down and say “Okay, let me think about what I can do better”. We probably should do that more, but that's hard to do.”

• “Before medical school, I didn't reflect very much. What I used as reflection was test scores. So, if I scored high then it must be a reflection of me doing well. Talking with my academic coach helped me reflect on the strategies I was using and what was working what wasn’t. I could take a step back and kind of get back on track.”

### Benefits of coaching: themes 6-8

#### Theme #6: improved wellbeing and quality of life

Coaching benefits students by allowing for time allocation to activities which enhance personal wellness, foster social relationships and support engagement in non-academic experiences in medical school.

• “I’ll just say that the quality of my life has improved significantly. The prep work took out too much time from all the other areas of my life. I was able to go home last week to see my family even though I have my second exam on Friday, so in terms of quality of life, I’m really happy I did this.”

• “…[academic coaching] made me much happier and much less stressed, like my quality of life returned from doing this work which has been huge, even if my actual academic achievement hasn't really changed.”

#### Theme #7: empowered to let go

Students express initial concern with not being able to read, learn, memorize or take notes on specific details of newly learned material. Having their academic coach verbalize the concept of being okay with not knowing everything was a reassuring experience.

• “I think everything was helpful, even the stuff that like I technically knew, like don't learn passively. I think just having someone tell me directly, “hey you don't need to know everything, you should just answer the questions and focus on the main points, you will actually remember more that way.””

#### Theme #8: bringing one’s best self to class

Students described benefit to using certain SRL skills in preparing for class and engaging in team-based classwork. This knowledge helped some to gain confidence in providing contributions to group discussions.

• “…I feel less of an added pressure because I realized, I could go to class, and I wouldn't be holding my teammates back because so much of what's in class is problem solving…so, I still felt like I was a contributing member of my team.”

• “In terms of how I relate to my classmates, it used to be that the first question would go up on the whiteboard and it was like my heart would race, like oh my gosh I have no idea what they're talking about and then it's like look back on the notes and hit command F. Since I’ve been doing the prep questions and using them as a guiding light it happens a little less, and now after coaching, the question will go up and I’ll be like okay, I know that they're asking me about this, I know that they want me to say, or they want me to look here.”

## Discussion

Participation in a self-regulated learning intervention resulted in increased motivation for the use of evidence-based learning strategies, goal setting, and reflection among first-year medical students. Through written responses and interviews, students indicated the educational benefit of SRL skill application and described a variety of methods for future integration of specific strategies into their learning routine. Encouraging and normalizing the practice of frequently reflecting on one’s learning strategies, areas for growth, and overall progress, irrespective of a students’ current level of academic achievement, may be beneficial, particularly during periods of academic transition.

Skills in SRL may be especially relevant in the context of active forms of learning such as problem-based learning and case-based learning. These methods require knowledge application and critical thinking [3, 21]. A study by Thomas et al. showed that students who actively engage in SRL strategies demonstrate increase in their use of metacognitive processes and express a greater sense of confidence in their approaches to learning in PBL [22]. While engaging in problem solving activities and collaborative learning with peers, students receive constant feedback regarding their level of preparedness for the class session and overall understanding of the material [5, 11, 15, 23]. To sufficiently improve course performance, continuous reflection, incorporation of feedback, and adaptation of learning skills are required [4, 24]. Our findings indicate that both classroom-based learning sessions and a one-on-one academic coaching program are feasible approaches for encouraging the use of self-regulated learning techniques. Shared benefits of the two formats may include the development of a common SRL vocabulary and mental model among students and faculty, in addition to the creation of protected time and space for reflection and goal setting. Table 2 demonstrates an interpretation of these key findings with elaborations of potential solutions offered by the classroom and coaching sessions.

**Table 2:** Potential Challenges in Self-Regulated Learning Skill Application and Potential Solutions Offered by SRL-Inspired Interventions

Challenge:	Classroom Intervention Solution:	Academic Coaching Intervention Solution:
● **Students discover that learning strategies which were effective in previous settings may now seem inefficient, particularly in active, team-based learning environments, but breaking learning habits can be difficult. In comparison to passive learning strategies (e.g. re-reading and excessive notetaking), the use of novel SRL skills may be associated with a learning curve and lack of instant gratification.**	● Faculty share literature and evidence-base supporting the long-term benefit of utilizing SRL skills and learning strategies. ● Students learn and discuss specific steps for implementation of active learning strategies (e.g. consolidation, self-quizzing) which may help improve efficiency and retention of material. ● Introducing the concept of self-regulated learning may encourage adaptability and openness to trying new and potentially more suitable ways of processing information for sustainable learning.	● Guided application and real-time practice of specific learning skills during coaching sessions inspires confidence for independent use of new evidence-based strategies. ● Students develop a tailored learning plan and set educational goals with their academic coach, allowing for identification of areas where efficiency may be enhanced with new study skills.
● **Students often recognize the value of reflection, goal setting, and using evidence-based learning strategies, however finding the time for engaging in these SRL skills is a limiting factor, especially when faced with large amounts of new information to learn.**	● Designated in-class session provides students with protected time and space for reflection and goal setting. ● Provision of reflection journals may decrease barriers to continued, routine reflection outside of class. ● Faculty explain the ways in which the course structure and curriculum are designed to support students’ ongoing application of effective learning strategies: ○ Interleaving of basic science topics ○ Integration of clinical knowledge into basic science courses ○ Incorporation or readiness assessments/pre-class quizzes	● Coaching sessions are guided by student reflections with the added benefit of external feedback. ● Discussing goals and areas for growth improve recognition of the ways SRL skills complement as opposed to detract from efficiency in learning in the long-term.
● **Focusing on areas of academic weakness may be psychologically challenging and students may develop feelings of uncertainty regarding the efficiency of their learning skills.**	● Guided discussions in small groups allows for discovery of shared challenges and experiences in both independent studying and classroom learning. ● Collaboratively brainstorming solutions to learning challenges in teams may encourage a system of peer support and understanding.	● Introducing important lifelong learning concepts including growth mindset, positivity in learning, and psychological safety may decrease anxiety and stress of identifying and addressing gaps in knowledge and areas for growth. ● Discussions with the academic coach allow for reassurance and normalization of initial discomfort with the learning adaptation process and transition to medical school.

The first column of the table describes common challenges faced by students in implementing self-regulated learning skills which may arise during the first year of medical school. The second and third column include elaborations of solutions offered by the classroom and coaching sessions respectively

Classroom-based SRL interventions may serve as an opportunity to improve students’ awareness of various active learning methods, their evidence-base, and appropriate application, while also engaging learners in small group discussions about shared challenges in building effective learning habits. Health professions students often hold misconceptions regarding various fundamental learning concepts. Piza et al. demonstrated that medical trainees endorsed often utilizing ineffective study habits which do not align with evidence-based principles [18]. Additionally, working in teams during the classroom session helps to foster a system of peer support and joint solution development. Peer group dialogues have been shown to benefit learners by encouraging reflection, and helping students navigate intra- and interpersonal challenges [25–27].

Academic coaching is a form of learning support which is firmly rooted in deliberate practice and SRL frameworks [19, 28–30]. Engagement in real-time practice with specific learning skills during coaching sessions helped students feel reassured in their learning adaptation capabilities. Cultivating a trusting relationship with the academic coach resulted in a source of validation and objective feedback for students. These findings align with a study by Wolff et al., in which students participating in a formal coaching program reported that the most successful components of the coaching experience included the coaches’ assistance with reflecting on, validating, and interpreting opportunities and challenges in order to determine next steps in learning. For these students, academic coaches also served important roles in their wellbeing [30]. We identified that the collaborative development of a learning plan during the coaching sessions invited for learner-led formation of educational goals and meaningful reflection among medical students. Similar results were found in a study of 171 learners enrolled in a longitudinal educational intervention in which medical students who received coaching, were significantly more likely to implement learning goals and incorporate performance feedback into their learning goals than those who did not [19].

There are several limitations of this study for consideration. In the post-intervention survey for the classroom component, we assessed students’ anticipated use of self-regulated learning skills, which may serve as a potential predictor of behavioral change, although whether this intention aligns with actual application and behavioral change is not known. It is possible however, that encouraging students to provide a written response and to elaborate on the specific ways in which they intend to apply each skill may increase the likelihood of behavioral change. Secondly, the process of obtaining a student sample for the coaching intervention likely introduced self-selection bias, with participating students potentially having an increased interest and motivation in utilizing SRL skills. Finally, many of the medical students majored in non-science topics or have taken several years off between their graduate and undergraduate studies, a format of training which differs from that of other countries.

Self-regulated learning is an ongoing process, thus future investigations regarding the potential benefit of implementing an SRL-inspired intervention during the preclinical setting in addition to the period of transition to the clinical setting may provide insight for the utility of longitudinal SRL skill enhancement.

## Supplementary Information

**Additional file 1.**

## Data Availability

The datasets generated and/or analyzed during the current study are not publicly available due to the private nature of students’ responses but are available from the corresponding author on reasonable request.
