# Supplementary material for: First-year medical students’ perceptions of a self-regulated learning-informed intervention: an exploratory study
Source: BMC Med Educ. 2022 Nov 29;22:821. doi: 10.1186/s12909-022-03908-4 (PMC9710124; doi:10.1186/s12909-022-03908-4)
Supplement: Supplementary file 1 — Additional file 1. [file 12909_2022_3908_MOESM1_ESM.docx]

**Supplementary Material**

**Contents:**

1. Pre-Class Intervention Self-Regulated Learning Survey
2. Post-Class Intervention Self-Regulated Learning Survey
3. Coaching Guides
4. Post-Coaching Semi-Structured Interview Guide

**Pre-Intervention Self-Regulated Survey:**

1. In the past, how frequently have you used goal setting in learning?

a. Everyday

b. Once a week

c. Once a month

d. Maybe once, but not regularly

e. I haven’t used goal setting yet.

1. Please describe one way you might advise a fellow medical student to use goal setting as a tool in learning.

1. In the past how frequently have you used evidence-based learning strategies?

a. Everyday

b. Once a week

c. Once a month

d. Maybe once, but not regularly

e. I haven’t used evidence-based learning strategies yet.

1. Please describe one way you might advise a fellow student to use an evidence-based learning strategy.

1. In the past, how frequently have you used reflection in learning?
2. Everyday
3. Once a week
4. Once a month
5. Maybe once, but not regularly
6. I haven’t used reflection in learning yet.
7. Please describe one way you might advise a fellow student to use reflection in learning.

1. What, if any, barriers do you anticipate may arise for a student attempting to use goal setting, reflection and/or effective learning strategies?

**Post-Intervention Self-Regulated Learning Survey:**

1. How frequently do you anticipate that you will use goal setting in your learning throughout the semester?
2. Everyday
3. Once a week
4. Once a month
5. Maybe at some point in the future.
6. I don’t anticipate using goal setting.
7. What is one way you could incorporate goal setting into your learning routine?
8. How frequently do you anticipate using evidence-based learning strategies throughout the semester?
9. Everyday
10. Once a week
11. Once a month
12. Maybe at some point in the future.
13. I don’t anticipate using evidence-based learning strategies.
14. What is one way you could incorporate effective learning strategies into your learning routine?

1. How frequently do you anticipate using reflection in your learning?
2. Everyday
3. Once a week
4. Once a month
5. Maybe at some point in the future.
6. I don’t anticipate using reflection.
7. What is one way you could incorporate reflection into your learning routine?

7. Are there any barriers which you might anticipate could interfere with your interest or ability to use goal setting, reflection and/or evidence-based learning strategies?

**Self-Regulated Learning Coaching Guides:**

**Coaching Guide Sessions 1:**

- 1. Establish relationship between the coach and coachee
  2. Define and discuss the concept of coaching and relation to SRL
  3. Explore students’ current academic and wellbeing status
     - Inquire about any conditions which may be impacting learning
  4. Define students’ goals for coaching program
  5. Learning plan development:
     - Guided reflection:
       - What is going well in learning process?
       - What are potential gaps in knowledge and learning skills?
       - How can the gaps be addressed? Initial learning goals?
     - Development of basic efficiency routine:
       - Before class: utilize preparatory questions
       - During class: engage in discussions, and fill in gaps from preparatory questions
       - After class: complete consolidation exercises
  6. SRL Learning skills and principles review
     - Discussion of evidence-based learning strategies
     - Course-specific learning principles
  7. Introduction to general psychological principles:
     - Growth mindset
     - Emotions and learning
  8. Practice Learning Skills
     - Based on learning plan, select learning strategy to practice (e.g. consolidation) using content from Foundations course

**Coaching Guide Sessions 2:**

1. Obtain student reflections and self-report
2. Discuss interim learning approach:
   1. Markers of progress?
   2. Learning skills applied?
   3. Learning challenges?
   4. Learning successes?
3. Provide objective feedback
4. Discussion of learning adjustments:
   1. Necessary adjustments?
   2. Why are adjustments needed?
   3. What will the adjustments look like?
5. Reintroduction of learning principles and strategies as necessary
6. SMART goal setting for remainder of the semester

**Post-Coaching Semi-Structured Interview Guide**

- Please tell me about your learning experiences thus far since starting medical school.
- Please tell me about your experience with the one-on-one academic coaching sessions.
- Please tell me about your motivation or reasoning for engaging in the one-on-one coaching sessions.
- What, if any, were your takeaways from your coaching experience?
- Please share if there have been any changes in your approach to learning in medical school since engagement with the coaching sessions?
- If you have any suggestions for how the coaching program may be improved, please share.
- Is there anything else which you would like to share about your experience?

Thank you very much for your time and participation.
